# The DiAMOND trial protocol: a randomised controlled trial of two decision aids for mode of delivery among women with a previous caesarean section [ISRCTN84367722]

**DOI:** 10.1186/1471-2393-4-25

**Published:** 2004-12-10

**Authors:** Alan A Montgomery, the DiAMOND Study Group

**Affiliations:** 1 Academic Unit of Primary Health Care, Department of Community Based Medicine, University of Bristol, The Grange, 1 Woodland Road, Bristol BS8 1AU, United Kingdom

## Abstract

**Background:**

Caesarean section (CS) has become an increasingly common method of delivery worldwide, rising in the UK from 9% of deliveries in 1980 to over 21% 2001. This increase, and the question of whether CS should be available to women on request, has been the subject of considerable debate, and national reports and guidelines have specifically highlighted the importance of patient choice in the decision making process. For women who have already experienced CS, the UK National Institute of Clinical Excellence recommends that the decision should consider maternal preferences and priorities in addition to general discussion of the overall risks and benefits of CS. Decision aids for many different medical treatment and screening decisions have been developed and evaluated, but there is relatively little evidence for the use of decision aids for choice of mode of delivery among women with a previous CS. The aim of the study is to evaluate two interventions to assist decision making about mode of delivery among pregnant women with one previous CS.

**Methods/design:**

Women with one previous CS are recruited to the trial during their booking visit at approximately 12–20 weeks' gestation in participating maternity units in Bristol, Weston and Dundee. Using central randomisation, women are allocated to one of three arms: information programme and website; decision analysis; usual care. Both interventions are computer-based, and are designed to provide women with detailed information about the potential outcomes for both mother and baby of planned vaginal delivery, planned CS and emergency CS. The decision analysis intervention additionally provides a recommended 'preferred option' based on maximised expected utility. There are two primary outcomes (decisional conflict and actual mode of delivery), and five secondary outcomes (anxiety, knowledge, perceptions of shared decision making; satisfaction with decision making process, proportion of women attempting vaginal delivery). Primary follow up for the questionnaire measures is at 36–37 weeks' gestation, and a total of 660 women will be recruited to the study. The primary intention-to-treat analyses will comprise three pair-wise comparisons between decision analysis, information and usual care groups, for each of the two primary outcomes. A qualitative study will investigate women's experiences of the decision making in more depth, and an economic evaluation from the perspective of the NHS will be conducted.

**Discussion:**

Provision of information to women facing this decision appears variable. The DiAMOND study aims to inform best practice in this area by evaluating the effectiveness of two interventions designed to aid decision making.

## Background

Over the last 20 years caesarean section (CS) has become an increasingly common method of delivery. The CS rate in the United Kingdom rose from 9% of deliveries in 1980 to 21% in 2001 [1]. This increase, and the question of whether CS should be available to women on request, has been the subject of considerable debate [2]. The optimal mode of delivery for women who have experienced a previous CS is complicated by the difficulty in balancing the risks of repeat CS with those of vaginal birth after caesarean section (VBAC). An evaluation of caesarean delivery by the American College of Obstetricians and Gynecologists reported that first time mothers with term singleton cephalic pregnancies and women with a previous CS account for two thirds of all caesarean deliveries in the US [3].

The Changing Childbirth report has emphasised the importance of patient choice when decisions need to be made in relation to the management of pregnancy and childbirth [4]. However the views of women who have experienced CS and their preferences for future deliveries have received little attention to date. Obstetricians tend to focus on the risks of uterine rupture and emergency caesarean section in labour [5], which may influence the advice they give to women uncertain about future mode of delivery. Others have focused on the increased morbidity following CS [6] and on the negative impact of operative delivery on first postnatal contact between the mother and her baby [7]. In Chile, where there is a very high rate of CS in the private sector, only a minority of women reported that they had wanted this method of delivery [8]. In a Scottish study, more women who delivered by elective CS reported they were satisfied with their involvement in the decision making process compared with women who underwent emergency CS [9]. The advantages and disadvantages of planned CS versus planned vaginal delivery has also been debated in north America in response to a growing number of requests for elective CS [10].

In an attempt to ensure appropriateness of CS in the UK, a set of evidence-based guidelines on indications for CS have recently been published [11]. The guidelines, commissioned by the UK National Institute for Clinical Excellence, make a specific recommendation that for women with a previous CS, the decision should consider maternal preferences and priorities in addition to general discussion of the overall risks and benefits of CS. It is essential that the process of decision making about future mode of delivery is evaluated and enhanced to achieve the safest and most satisfactory outcome for both mother and baby.

It has been proposed that the way in which clinical decisions are made lies on a continuum, from paternalistic (clinician decides) through partnership (clinician and patient share the decision) to informed (patient decides) [12]. Although proposed as the preferred approach of determining patients' treatments [13], some problems with the concepts, terminology and practice of shared medical decision making have recently been highlighted [14]. The appropriateness of the shared model may also depend on the clinical context as well as patients' and clinicians' preferences for involvement in decision making [15]. In addition, there is some evidence that patients and health professionals often have different treatment preferences, potentially making agreement on a treatment strategy more difficult [16].

Decision aids are designed to help people select between various treatment strategies by providing information on the options and outcomes relevant to a person's health. Decision aids for many clinical conditions have been developed [17], and evaluations of these decision aids have been the subject of systematic reviews [18,19]. A north American trial found no difference in terms of VBAC rate between written versus personal counselling interventions that actively promoted vaginal delivery [20]. An Australian trial of a paper-based decision aid for women who have previously experienced CS is currently underway [21].

The specific content of decision aids may vary, but in general they aim to present more than one strategy for clinical management, help people understand the probable outcomes of treatment choices and allow people to consider the personal value they place on benefits versus harms. Decision aids can take several formats, such as leaflets, interactive videodisks, individualised decision analysis, personal counselling sessions and audio workbooks. Interventions to assist patient decision making can improve knowledge about treatment options, make patients more realistic in their expectations, reduce decisional conflict and increase active involvement in decision making [18].

As an intervention to aid patient decision making, individualised decision analysis has so far received limited attention. By explicitly combining patients' values regarding treatment outcomes and individual probability information, decision analysis attempts to provide a rational framework to guide patient decision making. The use of individualised decision analysis has been debated [22], but empirical evidence demonstrates that it is feasible and acceptable and has value as an aid to patient decision making [23].

## Aim

The aim of this paper is to describe the protocol for a randomised controlled trial of two interventions to aid decision making about mode of delivery among pregnant women with one previous CS. The interventions being assessed are (1) Decision analysis, and (2) Information programme and website. Both decision aids will be compared with usual care given by the obstetric team. The interventions will be assessed in terms of decisional conflict, planned and actual mode of delivery, anxiety, knowledge, perception of shared decision making and satisfaction with the decision making process. Development and piloting of the interventions took place in 2003–2004, and the main phase of the study started in May 2004 and will continue until December 2006. Ethical approval for the study was obtained from the UK South West Multi-Centre Research Ethics Committee.

## Methods/design

### Recruitment and allocation of participants

The sample will comprise pregnant women with one previous lower segment CS (all parities will be included, but the most recent delivery must have been CS), no current obstetric problems and delivery expected at ≥ 37 weeks. Women are being recruited to the study by research midwives during their initial booking visit at approximately 12–20 weeks' gestation. Recruitment takes place from maternity units in St Michael's and Southmead Hospitals in Bristol, Weston General Hospital, and Ninewells Hospital in Dundee. The current CS rates for these units range between 18 and 24% and are representative of rates for other units throughout England and Scotland. The women are informed that although both vaginal delivery and repeat CS carry their own benefits and risks, the best method of presenting this information in order to assist women in reaching a decision is not known. Women expressing an interest in participating in the trial at the booking visit are given an information sheet, a written consent form and a baseline questionnaire to take home. Following receipt of the baseline questionnaire and written informed consent to enter the trial, women are randomised to one of three arms as detailed below. Allocation is stratified by maternity unit and preferred mode of delivery and blocked (using random permuted blocks of sizes 6, 9, 12 and 15) to ensure reasonable balance between the trial groups through time. The randomisation sequence was generated by a member of the study team (AAM), and allocation of participants is performed by a staff member with no other involvement in the study.

### Interventions

Both interventions are computer based. Women allocated to receive an intervention have an appointment with a researcher to allow the decision aid to be delivered using a laptop computer, usually in the woman's own home or workplace.

#### (i) Information programme and website

This intervention provides information about the outcomes associated with planned vaginal delivery, planned CS, and emergency CS. This includes descriptions of outcomes for both mother and baby, and the probabilities of these outcomes based on the best available evidence. Both the probabilities of having and not having the event are given, and all probabilities are presented in both numerical and pictorial format [24]. The programme easily allows women to choose the information that they view, and the information each women accesses is logged. At the end of the initial appointment with a researcher, women are given a password that allows them to access the information programme via the internet as often as they wish. Womens' use of the programme via the internet is also logged.

#### (ii) Decision analysis

There are generally four main steps involved in a decision analysis. The first is to draw up a decision tree that maps out the likely outcomes of the strategies in question [25]. These outcomes are then assigned utilities that represent how an individual values a particular outcome. A utility is a number between 0 and 1, often representing the outcomes 'death' and 'perfect health' respectively [26]. Probability information is then included in the tree to represent the chance of each outcome occurring [26]. Finally, strategies are compared by calculating the weighted sum of the utilities of all possible outcomes [27]. The recommended strategy is that with the highest expected utility value, or in other words, the one that gives an individual the best chance of achieving an outcome that is valued.

The decision analysis intervention in the trial proceeds according to the principles described above. First, women are given information about the outcomes associated with planned vaginal delivery, planned CS, and emergency CS. This includes descriptions of outcomes for both mother and baby, but not the probabilities of these events. These are embedded in the decision tree which is not visible to users. Second, women are required to rate (assign a utility value between 0 and 1) each possible outcome using a visual analogue scale. Finally, the programme combines the elicited utilities and the probabilities of each outcome in a decision tree to produce a recommended 'preferred option' based on maximised expected utility. Each woman is given a computer printout of the outcome of the decision analysis.

#### (iii) Usual care

This comprises care normally given by the obstetric and midwifery team. Women allocated to decision analysis or information programme receive these interventions in addition to usual care.

Women in both intervention arms are contacted by letter at 35 weeks' gestation. The purpose is to encourage discussion of the intervention with their obstetrician and/or midwife when they attend the clinic at 36–37 weeks to finalise their planned method of delivery. Participation in the study is recorded in the medical records of all women in the trial.

### Outcome measures

There are two primary outcomes:

(1) Decisional Conflict Scale (DCS) [28,29]. This is a 16 item questionnaire that measures degree of uncertainty about which course of action to take and the main modifiable factors contributing to uncertainty. Previous research indicates that effect sizes of about 0.35 to 0.5 standard deviations can discriminate between individuals who make a decision and those who delay or are unsure [28]. Assuming a standard deviation of 15 points [18], this is equivalent to differences of 5.25 to 7.5 points on the total DCS 100 point scale.

(2) Actual mode of delivery (vaginal, elective CS, or emergency CS). Unlike a previous trial [20], we are not seeking to promote one method of delivery over another. However differences between the arms in the proportions of different modes of delivery may have important healthcare resource implications.

There are five secondary outcomes: anxiety; [30] knowledge; perception of shared decision making; satisfaction with decision making process; proportion of women attempting vaginal delivery.

### Follow up

The primary and secondary outcomes are assessed in all three groups at baseline, and approximately two weeks after randomisation. The questionnaire at two weeks will constitute a secondary follow up, and will enable sufficient time for delivery of the appropriate interventions. As part of usual care, women in the trial normally attend the clinic at around 36–37 weeks' gestation to finalise plans for their preferred method of delivery. Questionnaire outcomes are measured again after this visit, and this constitutes the primary follow up for this trial. Actual and attempted mode of delivery (cross-checked with hospital records) and satisfaction with choice are assessed by a further postal questionnaire at approximately six to eight weeks after giving birth.

### Justification of sample size

As noted above, differences in excess of 0.35 standard deviations have been considered as important for the total DCS score, and differences of this magnitude are feasible for interventions of this kind [23]. With regard to mode of delivery, UK data indicate that about 33% of women with one previous CS are delivered vaginally [1]. The sample size calculation for a previous trial of written versus verbal counselling in north America presumed a vaginal delivery rate of 30% for a minimal intervention, and in the event observed that 51% of women achieved vaginal delivery for the trial groups overall [20]. A change from 30–33% to 51% corresponds to an odds ratio of about 2.1–2.4, and this would certainly be considered as clinically important.

With two-sided 1% alpha, a total sample size of 600 provides 82–99% power to detect a standardised difference of 0.35–0.5 in total decisional conflict score between the groups, and 84–95% power to detect odds ratios of 2.1–2.4 in women achieving vaginal delivery. A pair-wise alpha of 1%, corrected for multiple comparisons using Tukey's procedure, maintains an overall study-wise alpha of 3.4%. In order to allow for pre-term deliveries, malpresentations, and losses to follow-up, we will therefore recruit 660 women to the trial.

### Statistical analysis

Data analysis will proceed according to CONSORT guidelines for randomised controlled trials. The first stage of the analysis will be to use descriptive statistics to describe the group of individuals recruited to the trial in relation to those eligible, and to investigate comparability of the trial arms at baseline. The primary analyses will comprise three pair-wise intention-to-treat comparisons between decision analysis, information and usual care groups, for each of the two primary outcomes. These comparisons will use appropriate (that is, standard or logistic) multivariable regression models, adjusting for maternity unit, initial preference regarding mode of delivery, and value of the outcome variable at baseline. Full attention will be paid to the estimates and confidence intervals for these comparisons as well as the p-values, with the latter adjusted for multiple comparisons using Tukey's procedure. Secondary outcomes will then be analysed in the same way, using appropriate multivariable regression models depending on the nature of the outcomes.

Other secondary analyses will involve investigation of the short-term effects of the interventions using data from the two week follow up, and the effects at 36–37' weeks gestation adjusted for both baseline and two week follow up. Pre-planned subgroup analyses employing appropriate interaction terms in the regression models will be used to ascertain any differential effects of the interventions on the two primary outcomes across the following categories of women: previous caesarean section occurring before or after labour; previous successful vaginal delivery; stated preferred mode of delivery. Since the trial is powered to detect overall differences between the groups rather than interactions of this kind, the results of these essentially exploratory analyses will be presented using confidence intervals as well as p-values, and interpreted with due caution. Finally, we will investigate the effect of differential use of the information intervention via the internet using descriptive statistics and appropriate comparisons with the other groups.

### Qualitative study

Qualitative research methods will be used in order to explore aspects of the interventions and women's experiences of the decision making in more depth. Specifically, semi-structured interviews will be conducted with a sample of women from each of the intervention arms (Decision Analysis and Information), across the research sites in Bristol and Dundee. These interviews will explore:

(1) Women's views and experiences of the intervention and its delivery – for example, the quality and relevance of the decision aid/information, what they felt about the risk information presented to them, and which particular aspects of each intervention were helpful or unhelpful.

(2) Which factors women felt had most influenced their preferences regarding method of delivery.

(3) Whether the women had prior preferences about method of delivery, whether/how these changed during their pregnancy and in the case of the decision analysis, what they felt about the method of delivery proposed by the intervention compared to any prior preferences.

(4) Any other information sources women sought and used to help them make a decision about method of delivery (for example, information from health professionals, partner/family/friends, internet, media, books).

(5) Women's views and feelings about their actual method of delivery compared with any prior preferences and the method of delivery suggested by the intervention (for decision analysis).

A small number of interviews may also be conducted with women in the usual care arm of the trial to explore what support and advice was normally provided during pregnancy to those who did not receive an intervention.

A sample of approximately 30 women across the two intervention arms will be interviewed in depth to ensure a thorough exploration of emergent themes and concepts. Within each arm, women will be purposefully chosen to include those with different parities/ages/socio-economic backgrounds, and where possible different methods of delivery/outcomes, following a maximum variation sampling strategy [31]. The interviews will take place a short time after the primary follow up, to avoid any influence of the interview on these measures. A subset of the women will be interviewed a second time six to eight weeks after birth in order that they can reflect upon their decision regarding preferred mode of delivery with the actual mode of delivery. In addition, a small number of interviews may take place closer in time to receipt of the intervention if this is deemed (a) feasible given recruitment and (b) worthwhile in terms of providing new information to that gleaned from qualitative interviews undertaken in the development phase of the trial.

Interviews will be conducted in the womens' homes or other suitable setting chosen by them. A check-list of topics will be used to ensure that the primary issues are covered, whilst allowing flexibility for new issues to emerge from each interview. Interviews will be recorded on mini-disc, fully transcribed and anonymised to protect confidentiality. Transcripts will be studied in detail and a list of common themes and concepts drawn up. Data collection and analysis will run in parallel and the coding index added to or refined and coded material regrouped as new themes and categories emerge from subsequent interviews [32]. Further analysis will employ the constant comparison method of grounded theory in which the textual data is scrutinised for differences and similarities within themes keeping in mind the context in which mention of these themes arose in each interview [32].

In addition to the interviews with women, a small number of focus groups with health professionals (e.g. obstetricians, midwives) in each research site may be conducted near the end of the trial, resources and time permitting. Focus groups are often used in evaluations of new services/interventions and are useful for exploring group views, concerns and preferences (e.g. consensus or disagreement about an issue) [33]. They may be valuable for exploring professionals' views about the intervention and issues around implementation into routine practice.

### Economic evaluation

The aim of the economic evaluation is to compare the costs and benefits of the two interventions with usual care. The analysis will be from an NHS perspective and will be based on the costs incurred during pregnancy, delivery and 6–8 weeks following delivery. Incremental cost effectiveness ratios will be formed comparing (i) the cost per point improvement on the Decisional Conflict Scale (DCS) and (ii) the cost per extra patient able to make a decision (represented by a 0.5 standard deviation change on the DCS). We will also compare the average cost of care per patient in each arm of the trial with patient satisfaction. Differences in the cost of care of women receiving the two interventions will be compared with usual care from the point at which patients are randomised. The analysis will include all resources under the control of the NHS that may differ as a result of the interventions and will include resources used by both mother and baby. The costs identified as being of relevance are: antenatal appointments in addition to routine appointments, including both primary and secondary care; mode of delivery and related hospital stay for mother and baby; follow up care for mother and baby, including primary and secondary care appointments, outpatient appointments, inpatient stays, A&E visits, and prescribed medication.

Data on resource use will be collected principally by two questionnaires completed by the women. The first questionnaire, completed at 36–37 weeks' gestation, will provide information on all antenatal appointments, and the proportion of these that involved discussion about mode of delivery. Women will be asked how often mode of delivery was addressed at (i) routine appointments, and (ii) appointments initiated by them to discuss mode of delivery. A sample of 10 hand-held records from each arm at each main centre (St Michaels, Southmead and Ninewells, total 90) will be scrutinised to validate the information given in response to the questionnaire. The second questionnaire, completed six to eight weeks after delivery, will provide information on mode of delivery and all non-routine postnatal health service contacts and prescriptions for both mother and baby.

The principle of opportunity cost will underlie the valuation of resource use. In many cases, market rates will act as a proxy for opportunity cost. National data sets such as the Unit Costs of Health and Social Care [34] and the British National Formulary [35] will be used to value primary care consultations and prescribed medication. Secondary care contacts will be coded according to Healthcare Resource Group (HRG) and valued using the Department of Health National Reference Costs [36].

Costs and outcomes will not be discounted, as the economic evaluation will be limited to a period of 12 months. Sensitivity analysis will be conducted in areas where there is uncertainty around assumptions about resource use measurement and/or valuation. Variation in resource use and the effectiveness of the intervention is not captured by a cost-effectiveness/utility ratio. We will use bootstrapping to address this, and construct a cost effectiveness acceptability curve.

## Discussion

Women with an uncomplicated pregnancy and expected term delivery who have previously delivered by CS face a choice between repeat elective CS or attempted trial of labour. Guidelines in the UK emphasise the importance of involving women in the decision making process and taking account of maternal preferences and priorities, but the type and amount of information available to women facing this choice appears variable. The DiAMOND study is a randomised trial that aims to inform best practice in this area, by evaluating the effectiveness of two interventions to assist decision making in terms of decision quality and actual mode of delivery.

## Competing interests

The author(s) declare that they have no competing interests.

## Authors' contributions

Alan Montgomery, Deirdre Murphy, Ali Shaw and Sandra Hollinghurst drafted the manuscript, with input from the other members of the DiAMOND Study Group.

The Decision Aids for Mode Of Next Delivery (DiAMOND) Study Group comprises the following members: Clare Emmett, Tom Fahey, Peter Gregor, Sandra Hollinghurst, Claire Jones, Beverley Lovering, Alan Montgomery, Irene Munro, Deirdre Murphy, Roshni Patel, Tim Peters, Ian Ricketts, Anne Schlegelmilch, Ali Shaw, Kav Vedhara, Kate Warren.

## Pre-publication history

The pre-publication history for this paper can be accessed here:


