# Correction: The DiAMOND trial protocol: A randomised controlled trial of two decision aids for mode of delivery among women with a previous caesarean section [ISRCTN84367722]

**DOI:** 10.1186/1471-2393-6-1

**Published:** 2006-01-12

**Authors:** Alan A Montgomery, the DiAMOND Study Group

**Affiliations:** 1 Academic Unit of Primary Health Care, Department of Community Based Medicine, University of Bristol, The Grange, 1 Woodland Road, Bristol BS8 1AU, UK

In the economic evaluation section of the protocol [1], we stated that secondary care contacts would be coded according to Healthcare Resource Group (HRG). This has not been possible as HRGs are not routinely recorded in all of the recruiting hospitals, and have proved unreliable among the hospitals that do. Deliveries will instead be coded as 'normal', 'assisted', 'elective caesarean section', or 'emergency caesarean section' according to each patient's hospital record.

A further issue is that the Office of Scottish Health Statistics does not provide cost estimates for different types of delivery. Although costs in Scotland have been estimated using data collected in the early 1990s [2], we consider there to be substantial limitations in applying these costs to the current study. The economic evaluation will therefore apply English costs to the English data, and will investigate in sensitivity analyses the consequences of both applying English costs to, and excluding, the Scottish data.

## Pre-publication history

The pre-publication history for this paper can be accessed here:


